# Earthquake damage as a catalyst to abandonment of a Middle Bronze Age settlement: Tel Kabri, Israel

**DOI:** 10.1371/journal.pone.0239079

**Published:** 2020-09-11

**Authors:** Michael Lazar, Eric H. Cline, Roey Nickelsberg, Ruth Shahack-Gross, Assaf Yasur-Landau

**Affiliations:** 1 Dr. Moses Strauss Department of Marine Geosciences, Charney School of Marine Sciences, University of Haifa, Haifa, Israel; 2 Department of Classical and Near Eastern Languages and Civilizations, The George Washington University, Washington, DC, United States of America; 3 Department of Maritime Civilizations, Charney School of Marine Sciences, University of Haifa, Haifa, Israel; 4 Recanati Institute for Maritime Studies, University of Haifa, Haifa, Israel; Universita degli Studi di Milano, ITALY

## Abstract

For years there has been much speculation surrounding the abandonment of the Middle Bronze Age IIB palace of Tel Kabri, ca. 1700 BCE. There are no weapons, hoards of money and jewelry, or visible evidence for fire, which rules out hostile attack or conquest. There are also no indications of drought or environmental degradation that might have forced the inhabitants to vacate the site, nor mass graveyards to indicate a pandemic. The current study uses micro-geoarchaeological methods to show that the demise of the palace was rapid, with walls and ceilings collapsing at once prior to abandonment. Macroscopic data (stratigraphic and structural) from five excavation seasons were reexamined, showing that at least nine Potential Earthquake Archaeological Effects (PEAEs) are found and associated with the last occupation phase of the site’s palace. All lines of evidence point to the possibility that an earthquake damaged the palace, possibly to a point where it was no longer economically viable to repair. This conclusion is compounded by the discovery of a 1–3 m wide trench that cuts through the palace for 30 m, which may be the result of ground shaking or liquefaction caused by an earthquake. This study shows the importance of combining macro- and micro-archaeological methods for the identification of ancient earthquakes, together with the need to evaluate alternative scenarios of climatic, environmental, and economic collapse, as well as human-induced destruction before a seismic event scenario can be proposed.

## Introduction

**Fig 1:**
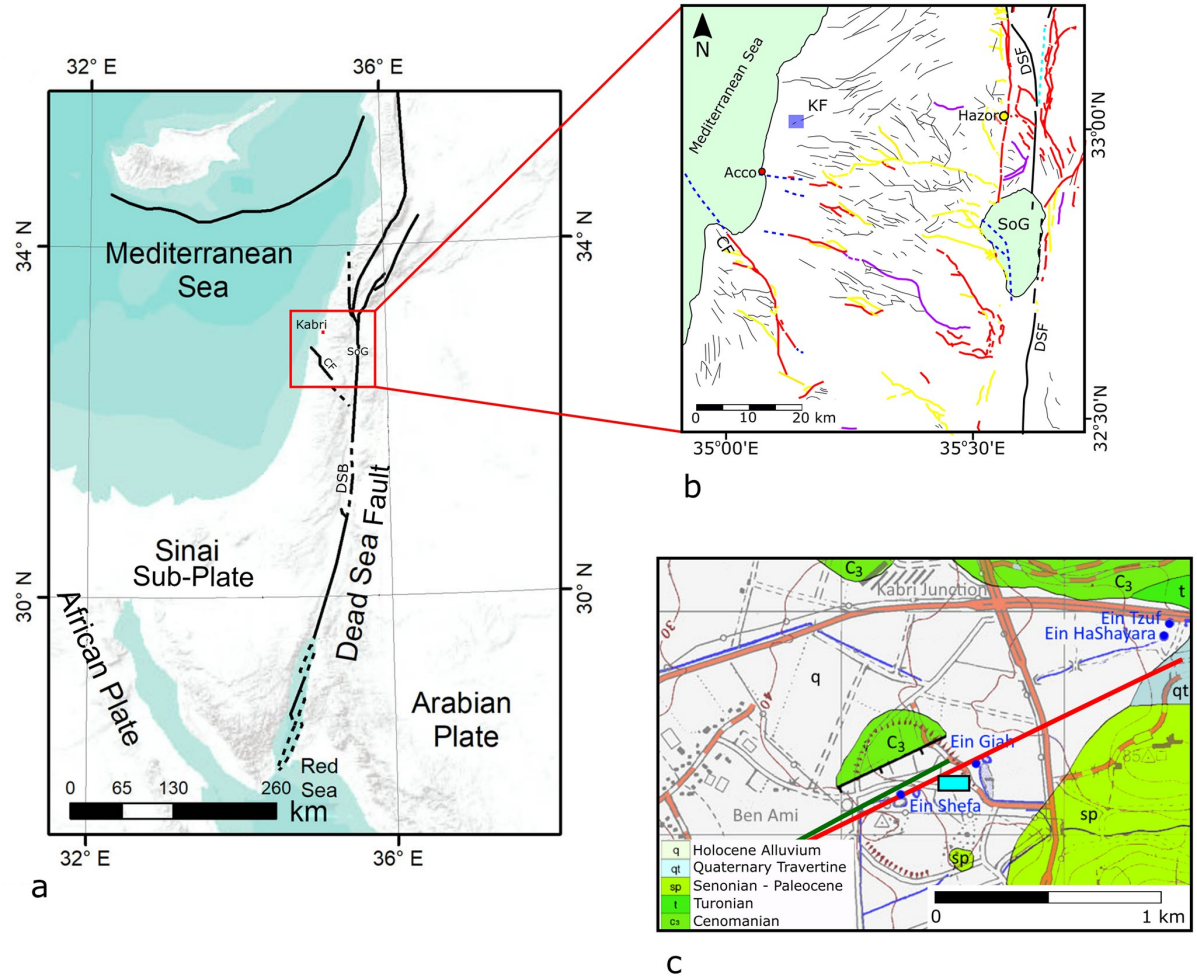
Tectonic setting of study area. (a) General map showing the main tectonic features in the eastern Mediterranean. SoG—Sea of Galilee. CF—Carmel fault. DSB—Dead Sea basin. Red dot marks the location of Tel Kabri. Reprinted from [21] under a CC BY license, with permission from [Copernicus Publications], original copyright [2019]. (b) Compiled map of faults in the Galilee area. Thin black lines indicate faults that appear on the 1:200000 geological map (Reprinted from [22] under a CC BY license, with permission from [Geological Survey of Israel], original copyright [1998]). Colored lines mark Quaternary faults (Reprinted from [23] under a CC BY license, with permission from [Geological Survey of Israel], original copyright [2018]): Red—evidence of Quaternary activity; Yellow—marginal faults and main branches; Purple—> 6km segments associated with recent activity; Blue dashed—inferred/subsurface; Thick black—Main strike-slip segments of the Dead Sea fault (DSF). Blue square marks location of geological map shown in Fig 1c. SoG—Sea of Galilee. CF—Carmel fault. KF—Kabri fault. Red circle marks the location of Acco, while the yellow circle marks the ancient city of Hazor. (c) Geological map of the area (Reprinted from [22] under a CC BY license, with permission from [Geological Survey of Israel], original copyright [1998]) showing the mound of Tel Kabri and its associated potentially active tectonic fault. Colored lines mark different interpretations for the Kabri fault: black line after [22]; green line after [24]; red line after [25]. Blue dots mark the location of the four springs located in the vicinity of Tel Kabri. Light blue rectangle marks the location of the study area shown in Fig 2.

Recognizing past earthquakes can be extremely challenging in the archaeological record, especially at sites where stone masonry is meagre and use of degradable construction materials such as sun-dried mudbricks and wattle-and-daub was practiced. Therefore, the effect of earthquakes on the archaeological record remains debated and often viewed as “catastrophism” (e.g., [1–11]). Recent studies have applied an integrated approach for identifying ancient earthquakes, combining the location of faults with the expected effects of earthquakes on site stratigraphy, structures, and artifacts found within sites and observable by the naked eye [11–18]. The term "Potential Earthquake Archaeological Effects" (PEAEs) has been used in such instances, when discussing the effects of earthquakes on archaeological sites [e.g., 11]. However, these macroscopic indicators can be further enhanced by the inclusion of supporting evidence from microscopic techniques routinely used in modern geoarchaeology, such as micromorphology [19] and microarchaeology (*sensu* [20]). These approaches are referred to here collectively as ‘micro-geoarchaeology'. Combined, macro-indicators for earthquakes and micro-geoarchaeology can help examine different modes of destruction and abandonment, and provide a wider spectrum of criteria than applied in previous studies. Here we present such an integrative study, identifying macro- and micro-indicators for one or more earthquake(s) that appear to have damaged the palace at the site of Tel Kabri in northern Israel (Fig 1) ca. 1700 BCE.

[26] first tentatively suggested that an earthquake may have been responsible for part of the damage observed in the palace at Kabri. However, continued research has now contributed additional information on issues related to destruction and abandonment. The site of Tel Kabri is examined here in light of these findings.

### The site

Tel Kabri is a 34-hectare site located in the western Galilee, Israel. Its location on a large mound overlooking the floodplain of the Ga’aton Stream made it favorable to human habitation, with an abundant water supply and soil for cultivation. The region is characterized geologically by Upper Cretaceous marine carbonate rocks on the east. The Ga’aton stream drains Cenomanian dolomite and limestone, Turonian limestone, and Senonian chalk and marl westwards to the Mediterranean Sea. Chert nodules occur in some rock formations [27]. Terra Rossa soil prevails on limestone and dolomite terrains and thick alluvial soils (grumusol) characterize the valleys [28]. The site was constructed around two springs, with at least two more in the immediate vicinity ([29]; Fig 1). All are fed by karstic limestone and dolomite aquifers from the east [30].

Tel Kabri flourished during the Middle Bronze Age (hereafter MB) and was the third largest site in the Levant at the time (after Hazor and Ashkelon). It was a fortified center of a regional polity and housed the largest MB palace found to date in the southern Levant, with an estimated area of 6000 m^2^. The palace is known from its modest beginning in the MB I (Kabri Area D-West, stratigraphic Phase VI) to its zenith in the MB II (Kabri Area D-West, stratigraphic Phase III) [31–33], dated ca. 1900–1700 BCE (high middle chronology [34]; cf. [35]).

During its final phase, the palace underwent massive renovation, reaching its greatest size. This involved the addition of a two-room complex lined with carved stone blocks known as “orthostats” (the “Orthostat Building”), probably used for banqueting, and a wing for the accommodation of hundreds of large storage jars (pithoi) containing spiced wine—the “Southern and Northern Storage Complexes” [26, 31–33, 36].

At the end of this phase, ca. 1700 BCE, the palace and its surrounding areas were abandoned [34], for reasons that are still unclear. The site then lay uninhabited for almost a millennium, after which only minimal human activity is recorded from the Iron Age and later (e.g. [31] and references therein). This study examines the possibility that the demise of this palace and settlement, during a period of flourishing and expansion, may be attributed to an earthquake.

### Neotectonic background

#### The Dead Sea fault

When examining possible earthquake scenarios, it is important to understand the regional and local (neo)tectonic setting. The study area is located ca. 40 km west of the Dead Sea fault (DSF)–a left-lateral strike-slip plate boundary separating the Arabian plate from the Sinai sub-plate (Fig 1a). The DSF is the most tectonically-active feature in the area and most of the earthquakes occurring in the southern Levant are assumed to have originated along its trace. Analysis of these earthquakes over the years has led to the conclusion that they can cause significant damage far from the main fault trace (e.g. [21, 37, 38]).

In terms of historic earthquakes, the Dead Sea basin, one of the pull-apart basins located along the length of the DSF, preserves in its sediments the largest and most comprehensive near-continuous record of earthquakes in the southeastern Levant going back at least 70,000 years (e.g. [39–41]). Examination of these records for a possible large earthquake at the time of the damage of the Tel Kabri palace indicates the occurrence of an earthquake around 3700 BP (i.e., 1700 BCE). However, there is little evidence to connect this event with the destruction at Tel Kabri.

More recent historical accounts indicate that past earthquakes have had devastating consequences in the proximity of Tel Kabri. One example of this can be found in the August 21^st^ 502 AD M_L_ 7 event that totally destroyed the city of Acre (Acco) some 10 km to the southwest of Kabri (e.g. [42, 43]; Fig 1b). In this case, the epicenter is thought to have occurred offshore of Acco [42]. By contrast, the May 20, 1202 M_L_ 7.5 earthquake is thought to have destroyed at least one third of Acco and originated along the Dead Sea fault in Lebanon [43]. Both earthquakes are recorded in the sediments of the Dead Sea [40] and most likely would have caused great damage in the vicinity of Kabri as well, were anybody there to report on it.

Despite the presence of contemporary archaeological sites near Kabri, such as Tel Hazor and Acco (Fig 1b), there have been no reports from these extensively excavated sites of evidence for MB II earthquakes. This could be due to a number of factors, such as the difficulty in recognizing the expression of ground shaking due to earthquakes in structures that were built of mudbrick.

#### The Kabri fault

A number of potentially active faults can be found in the vicinity of Tel Kabri (Fig 1b). However, the site is located along the trace of a NE-SW trending normal fault (Fig 1b and 1c), termed the Kabri fault (e.g., [24, 44]). The Kabri fault is part of a 10 km long fault that stems from the foothills of the Galilee mountains, across the coastal plain towards the Mediterranean coast of northern Israel. It is one of a network of normal faults in the Galilee region [45]. Little-to-no work has been conducted along the trace of the Kabri fault. Marked on geological maps, it has been thought to be potentially active [25, 46]. This activity is evident by the series of springs that lie along its trace [29, 44]. According to [47], Tel Kabri is located within an area of anomalously high potential ground motion amplification; i.e., the site is susceptible to amplified ground shaking during an earthquake. Other nearby MB sites, such as Hazor, which is not located in such an area, do not seem to be prone to amplification.

The aim of this study is to assess the possibility that Tel Kabri was affected by a destructive earthquake that damaged the palace and its surroundings to a point that caused its complete abandonment. Our approach is integrative, combining macroscopic data in the form of PEAEs with micro-geoarchaeological evidence, further supported through an examination of a variety of other possible causes for destruction and abandonment (elaborated on in the Discussion).

## Methods

All necessary permits were obtained for the described study, which complied with all relevant regulations. Excavation permits were given by the Israel Antiquities Authority to the excavators (AYL and EHC), permit nos. G-52/2017 and G-16/2019. The land accessed is owned by Kibbutz Kabri. However, the Tel is on the list of antiquity sites of the Israel Antiquities Authority.

### Macro-archaeological indicators (PEAEs)

All observations related to architectural features and artifacts were routinely recorded by field measurements, photography, excavation plans, and post-excavation reports during excavation seasons that took place between 2011 and 2019. Criteria for the identification of off-fault earthquake damage [17], i.e., PEAEs, are based on [11], who adjusted these to sites characterized by rubble and mudbrick architecture such as that found in MB II Kabri (Table 1). According to their study, aside from the main structural indicators such as folded, rotated, or tilted walls, there are seven additional primary (direct) criteria that can be used to identify off-fault earthquake damage [17] in mudbrick constructions. These include compact layers of rubble suggesting wall collapse; folded/faulted floor structures and archaeological deposits; pockmarked floors; localized fire damage; broken, in situ vessels; broken, fallen vessels from furniture or upper floors; and oriented fallen objects.

**Table 1:** Potential (off-fault) Earthquake Archaeological Effects (PEAEs) (after [11]) and corresponding findings in Kabri.

PEAEs	Findings at Kabri	Location at Kabri
Compact layer of rubble burying valuables suggesting sudden collapse	Fill deposits characterized by massive structure and chaotic fabric, composed of mud brick material and plaster fragments. No micromorphological indications for slow/gradual accumulation of the deposit	Deposits directly above the floors of the Orthostat Building and Southern Storage Complex
Folded/faulted floor surfaces and archaeological deposits	Damaged and warped floors	Orthostat Building, Southern Storage Complex (Room 2440)
Earthen and plaster floor surfaces pock-marked by collapsed material	Damaged and cracked floors	Orthostat Building; Northern and Southern Storage Complexes
Localized fire damage	No evidence	N/A
Broken *in situ* vessels	Broken wine jars	Northern and Southern Storage Complexes
Broken, fallen vessels from upper floor	Spindle whorls and loom weights; possibly also vessels	Across the site in presumed second story collapse layers
Oriented fallen objects	Storage jars	Northern and Southern Storage Complexes
**Earthquake effects expressed in architectural elements**		
Tilted walls	Threshold leading into back room of Orthostat building tilted into trench; Wall of Northern Storage Complex tilted into trench	Orthostat Building; Northern Storage Complex
Displaced walls	Displaced orthostats, offset and/or wavy walls	Northern and Southern Storage Complexes; Orthostat Building

### Micro-geoarchaeological indicators

Sediments belonging to Phase III deposits and features were sampled based on stratigraphic and contextual considerations. The stratigraphy of this phase includes two beds: the Phase III plaster floor and a homogenous, gravelly, greyish-brown deposit above it. As no finer bedding was observed macroscopically in the field, samples of the floor and homogenous fill above it were collected randomly from exposed sections. In addition, samples of mud bricks from a mudbrick wall stump, as well as from its thin plaster lining, were collected to serve as reference to MB II construction materials, likely representing the natural sediments that were available near the site during the Middle Bronze Age. Sediments were sampled in two formats: undisturbed blocks for micromorphology and loose (bulk) for mineralogical analyses.

In terms of micromorphology, mudbrick block samples were obtained from wall 2450 of Phase III (n = 3), while the floor of Phase III and the deposits directly above it were sampled in block format in two locations across the Southern Storage Complex (S1 Fig and S1 Table).

The undisturbed sediment blocks were placed in an oven (Binder) at 50°C for a few days. The dried blocks were then impregnated with a mixture comprised of seven parts polyester and three parts acetone (v:v) with 10 ml MEKP hardener for every liter of mixture. The impregnation process was done in a fume hood, allowing slow polymerization of the resin for about one month. The hardened blocks were placed in the oven at 50°C for a two day curing period, then cut by a Hillquist RF 20–24 Slab Saw. Thin sections (5x7 cm, 30μm thick) were prepared by Quality Thin Sections (Arizona). Micromorphological examination and description were conducted using a polarized light microscope (Nikon Eclipse 50i POL) under plain polarized light (PPL) and cross polarized light (XPL) at magnifications between 25 and 400X. Descriptions of thin sections follow the terminology given in [48].

In terms of mineralogy, bulk sediment samples were collected from the mudbrick wall as well as from deposits exposed along profiles of Phase III (S1 Fig and S2 Table). Sampling from profiles was based on differences in sediment color as well as contextual position (i.e., directly on the Phase III floor or above it).

Bulk samples were air-dried and FTIR spectra were obtained following grinding of a small portion of the sample. They were then homogenized with potassium bromide (KBr), at a ratio of about 1:20 sample to KBr, using an agate mortar and pestle and pressed into a 7mm pellet (see details in [49]). The pellet was placed in a Nicolet iS5 FTIR spectrometer and spectra were collected between 4000 and 400cm^-1^ at 4 cm^-1^ resolution. The spectra were interpreted in reference to guidelines about the effect of heat on clay minerals [48] and the effect of heat on calcite [50].

## Results

### Stratigraphic and depositional macro-indicators (PEAEs)

**Fig 2:**
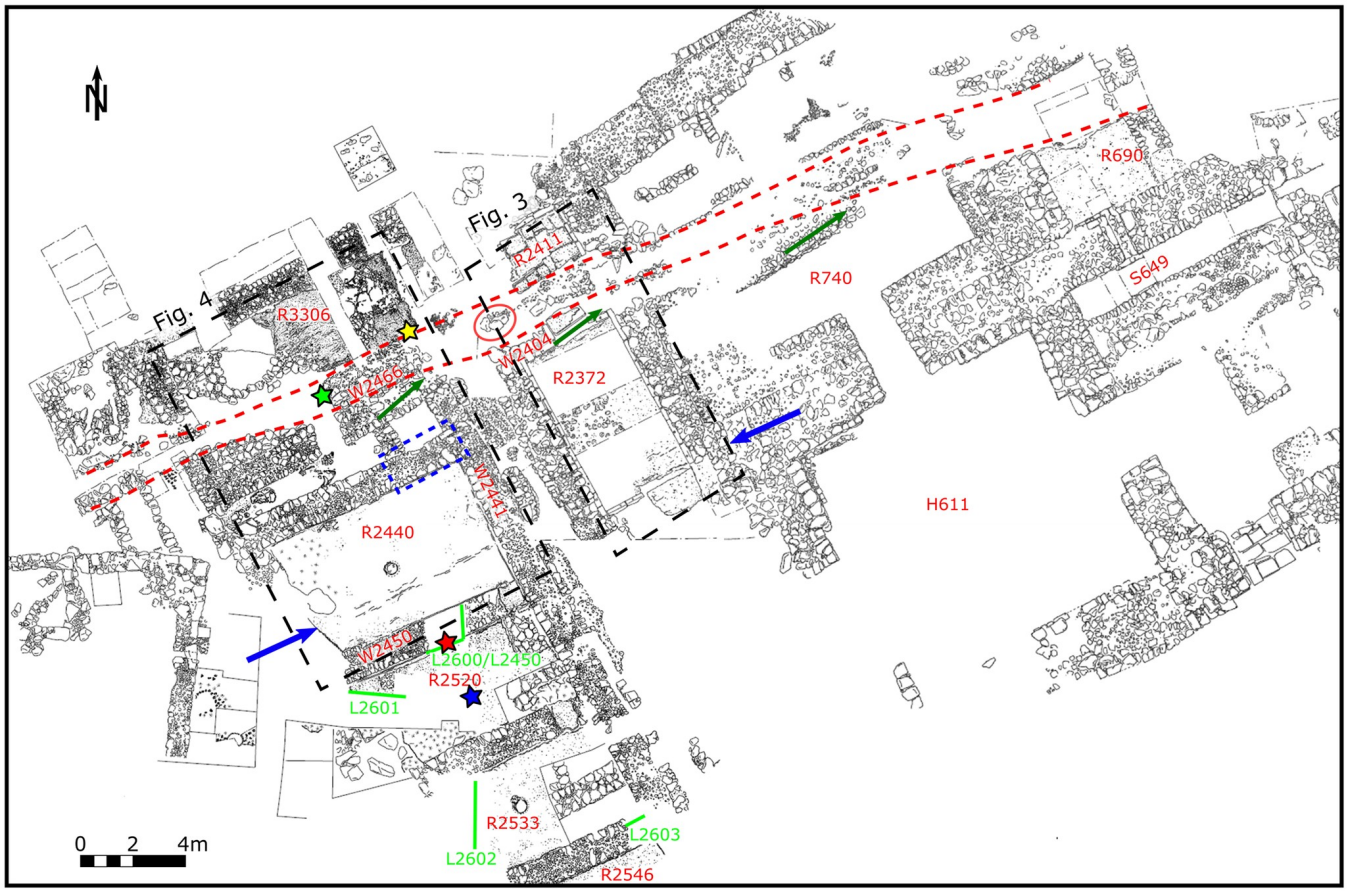
Plan of Phase III at Tel Kabri. Text starting with R indicates room numbers mentioned in text; W indicates wall locus numbers. Red circle indicates the location of an Iron Age pit, while red dashed line is the trace of the trench mentioned in the text. Green numbers beginning with L indicate loci where sediment samples were collected for micromorphology and FTIR analysis and green lines show the location of excavation profiles sampled. Blue arrows point to the warped floor in Room 2440 and offset walls of the Orthostat Building, which are aligned and parallel to the trace of the Kabri fault and to the trench. Note that structures located just south of the trench are misaligned with respect to the general trend of palace walls, with their northwestern corners rotated towards this feature (dark green arrows). Green star marks the approximate location of Fig 5a. Yellow star marks the approximate location of Fig 5b. Red and blue stars indicate the location of images presented in S1 Fig. Dashed black rectangles show location of Fig 3 (the Orthostat Building) and Fig 4a (the Southern and Northern Wine Storage Complexes), while dashed blue rectangle marks the location of Fig 4d.

The structures mentioned below belong to the end of Phase III, the time when the palace and surroundings were destroyed and abandoned (all locations correspond to rooms and walls in Fig 2). Each sub-section refers to one of seven Potential Earthquake Archaeological Effects (PEAEs) defined by [11] (Table 1).

#### Compact layer of rubble burying valuables suggesting sudden collapse

**Fig 3:**
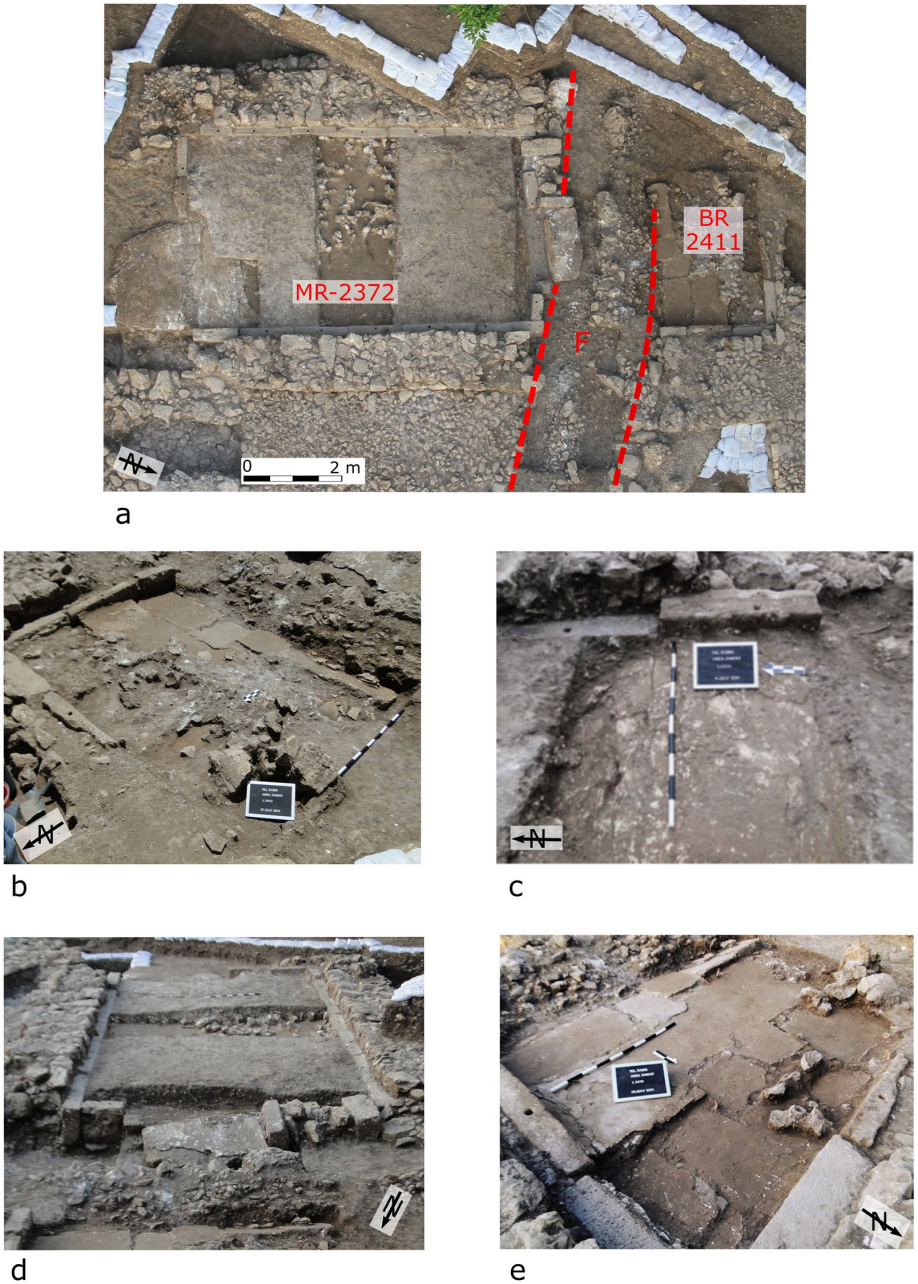
The Orthostat Building. Each black or white segment on the scale-sticks represents 10 cm. (a) Aerial view showing the Main Room (MR), Back Room (BR), and Trench (T), which is denoted by dashed red lines. Notice that the southern side of the trench is partially obscured by a large stone block, whose flat upper side would have once served as a threshold leading into the back room, but which has now collapsed into the trench. Photo: Griffin Aerial Imaging. (b) The backroom (2411) covered in collapsed mudbrick and plaster. (c) Step or displacement in the floor of the Orthostat Building, just inside the entrance. One of the offset orthostats is visible at the top of the image. (d) The Main Room looking south and showing unbroken warped cross-walls; i.e., with a rise visible in the height of the walls. At the bottom of the image, wall 2404 and the threshold leading into the back room are visibly collapsed and tilting into the trench. (e) The backroom cleared of all rubble shown in Fig 3b, showing the broken and sloping plaster floor.

The entire floors of the main and back rooms of the Orthostat Building (Rooms 2372 and 2411 respectively; Fig 3a) were covered by a ca. 30 cm thick layer, composed of sizeable pieces of plaster and mudbrick (Fig 3b). This suggests that the deposit is the result of the collapse of the ceiling and walls. In addition, all the rooms of the Storage Complex were covered by a 42–67 cm thick layer of collapsed mudbrick and plaster from either the ceiling or the walls (e.g. Room 2440; Room 3306), as also shown micromorphologically. These collapse deposits cover valuables such as wine-containing pithoi, the contents of which could have been siphoned off and removed in the event of a slower abandonment of the site, had that happened (see below).

#### Folded/faulted floor surfaces and archaeological deposits

The orthostats comprising the western and eastern walls of the main room of the Orthostat Building (Room 2372) are aligned, except for those at the southern end of both walls, near the entrance. These are elevated by 15 cm (Fig 3c). This change in elevation is also apparent in the floor level of the room, which slopes down (proceeding north from the room entrance) by 20 cm in less than half a meter, beginning at the spot where the orthostats change in elevation (Fig 3c). This deformation of the floor just inside the entrance is very unlikely to have been a feature existing prior to the destruction, since it would have made the floor surface irregular and rather unusable. To the north, the floor remains relatively flat, dropping only 2 cm over a distance of 6 m.

The northern wall (W2404), with a threshold originally leading into the back room, is heavily deformed and has collapsed northward into the trench (Fig 3d), which now separates the main room from the back one (Room 2411). Elevation of the orthostats in the latter is 4–5 cm lower than in the main room and gaps of ca. 2 cm appear between these and the stone walls.

**Fig 4:**
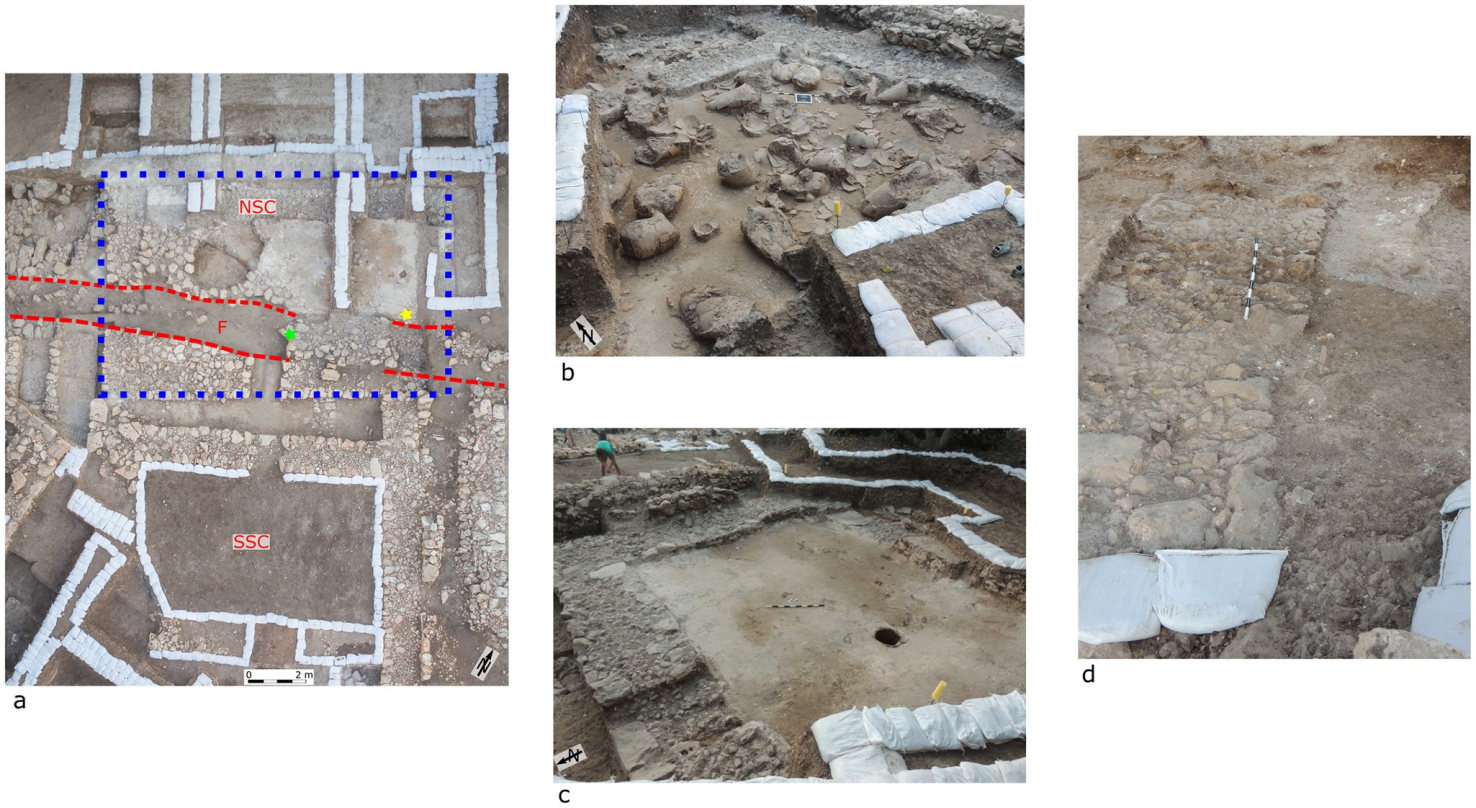
The wine cellar (Room 2440 in the Southern Storage Complex). (a) Aerial view showing the Southern Storage Complex (SSC), the Northern Storage Complex (NSC; blue dashed box) and the Trench (T; also denoted by the dashed red lines). Green star marks the approximate location of Fig 5a. Yellow star marks the approximate location of Fig 5b. Photo: Griffin Aerial Imaging. (b) Room 2440 showing ~48 pithoi found in situ. Note the warping in the eastern wall of the room (W2441 Fig 2). (c) Room 2440 after being cleared of the pithoi. (d) Displaced wall W2443 in Room 2440 marked by blue dashed box on Fig 2.

In contrast, the plaster floor of the back room, now located on the far side of the trench, is broken up and slopes southward into the trench (Fig 3e). Similarly, within the Northern Storage Complex located just to the west(Fig 4a), the plaster floor also slopes to the south, into the large trench, and exhibits as well a clear fold (Room 3306; Fig 4b and 4c).

#### Pockmarked floor

Both the earthen and plaster surfaces of the Orthostat Building and the Storage Complex exhibit evidence for impact marks from collapsed vessels, construction debris, and objects that have fallen from above.

#### Localized fire damage

Localized fire damage was not evident in the field, nor was it apparent using the micro geoarchaeological indicators (below).

#### Broken, in situ vessels

The back room of the Orthostat Building included many pottery sherds that were smashed in situ [26] (Fig 3b). The pithoi in the rooms of the Storage Complex were found lying in position where they fell. All were smashed in place and most were crushed to some degree, though some in Room 2440 retained their shape (Fig 4b; [33, 36]).

#### Broken, fallen vessels from furniture or upper floor

Although it is unclear whether any vessels had fallen from an upper floor, loom weights were found in several concentrations in Room 690, the adjacent Room 740, and Stairwell 694, in collapse layers above the floors. They belonged to at least two looms that were likely located in a second story room [51].

#### Oriented fallen objects

In Room 2520, approximately twenty pithoi were found, most lying with an east-west orientation. A similar situation was found just to the south in Room 2533, where the bases of most of the thirty pithoi and other vessels found there were facing west. The eight vessels that were found in the small area that was exposed in the southernmost room (Room 2546) roughly adhere to this orientation pattern. Some 22 additional storage jars were recovered from the Northern Storage Complex, most of which were concentrated in the eastern and southern parts of the room.

#### Macro-indicators—Conclusion

The Potential Earthquake Archaeological Effects (PEAEs) presented above support six of the seven primary criteria set by [11] as evidence for potential earthquake identification. These adhere to off-fault effect as defined by [17]. This is in addition to other structural indications, such as tilted and folded walls, which were documented in the field (e.g. Fig 4d).

### The trench

**Fig 5:**
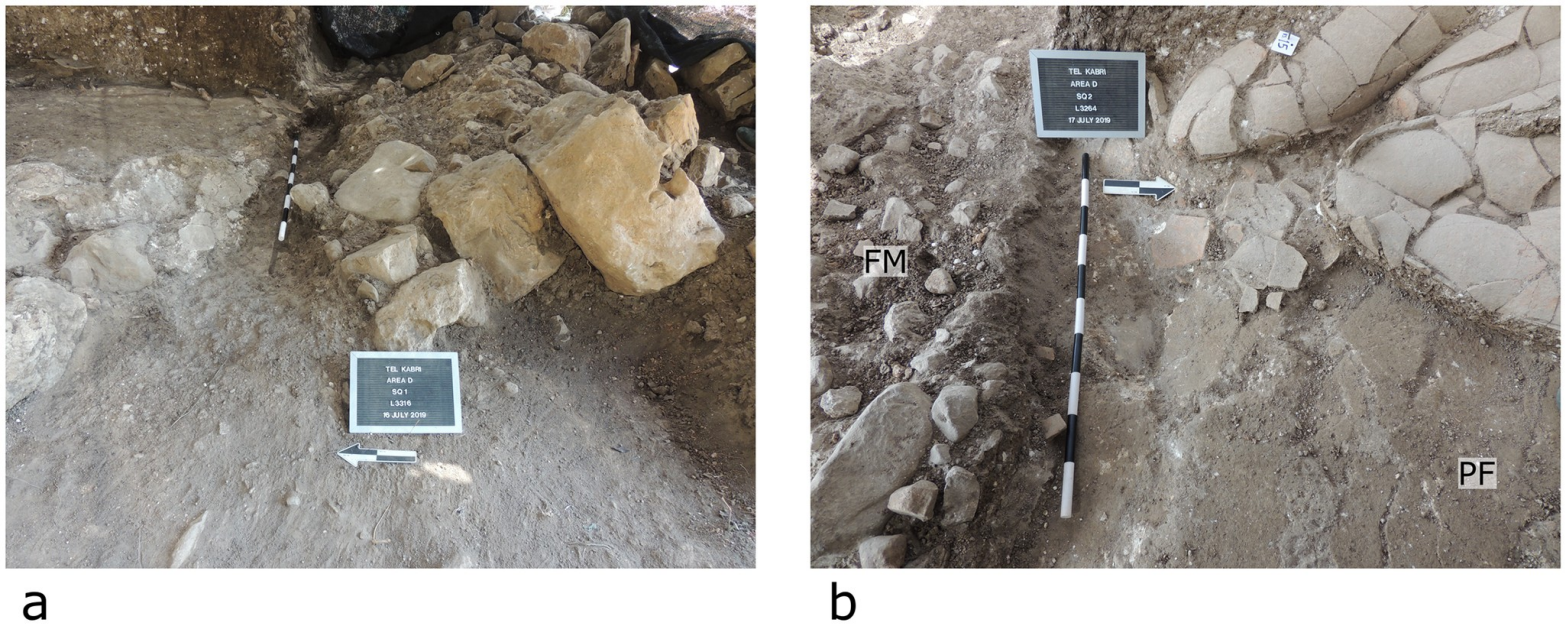
Field photos of collapse into the trench. (a) Photo looking northeast showing the collapse of Wall W2466 into the trench, which is now blocked by it. Arrow indicates north. (b) Photo from above in Room 3306 of the Northern Storage Complex. Left side of the image shows the trench. Right side of image shows pithoi that appear to have rolled southwards towards the trench and the collapse of the plaster floor into this feature. Arrow points to north. Each section of the scale-sticks corresponds to 10 cm.

Both the Orthostat Building and the Storage Complex show signs of having been cut by what was thought at the time of initial excavation (2011–2017) to be a modern-day trench. However, careful reexamination, together with finds from the Northern Storage Complex made during the 2019 season, suggest a very different story. This is a 1–3 m wide feature, which runs diagonally from southwest to northeast for at least 30 m. It is remarkably similar in angle and in strike to the Kabri fault line (Figs 1c and 2). The northern-most wall of the Southern Storage Complex (Wall 2466) collapsed into this feature, remains of which (three courses) were found slanting to the north (Fig 5a). Similar sloping was identified in the northern part of the Orthostat Building, where the threshold leading into the back room fell into the feature and is now lying at a steep south-to-north angle (Fig 4a and 4d).

On the other side of the trench, i.e., to the north, within the Northern Storage Complex, Room 3306 contains mudbrick collapse and a number of vessels that are concentrated in the southeastern part of the room (Fig 5b). The floor slopes to the south, i.e. towards and into the trench. Interestingly, while it cannot be conclusively proven, some of the vessels appear to have rolled to the edge, where they were found on their sides adjacent to one another, with others falling on top of them (Fig 4d). The plaster floor in the back room of the Orthostat Building also slopes to the south, towards the trench, as mentioned above (Fig 3e).

In general, analysis of archaeological finds in the field indicate that the remains to the north of the trench fell or now slope southward into it, while the remains to the south fell or now slope northward into it. These observations indicate that the trench is stratigraphically later than the construction of this part of the palace.

### Micro-geoarchaeological indicators

Micro-geoarchaeological data were examined by methods of micromorphology and FTIR spectroscopy, the former to study the mode of sediment accumulation on the last floor of the palace while the latter was used to determine whether heat was involved in the palace’s demise. Sediment samples included deposits above the floor belonging to the last phase of the palace, while sun-dried mudbricks preserved in a wall segment served as a control. Samples and their location are presented in Fig 2 and S1 Fig, S1 and S2 Tables.

#### Micromorphology

**Fig 6:**
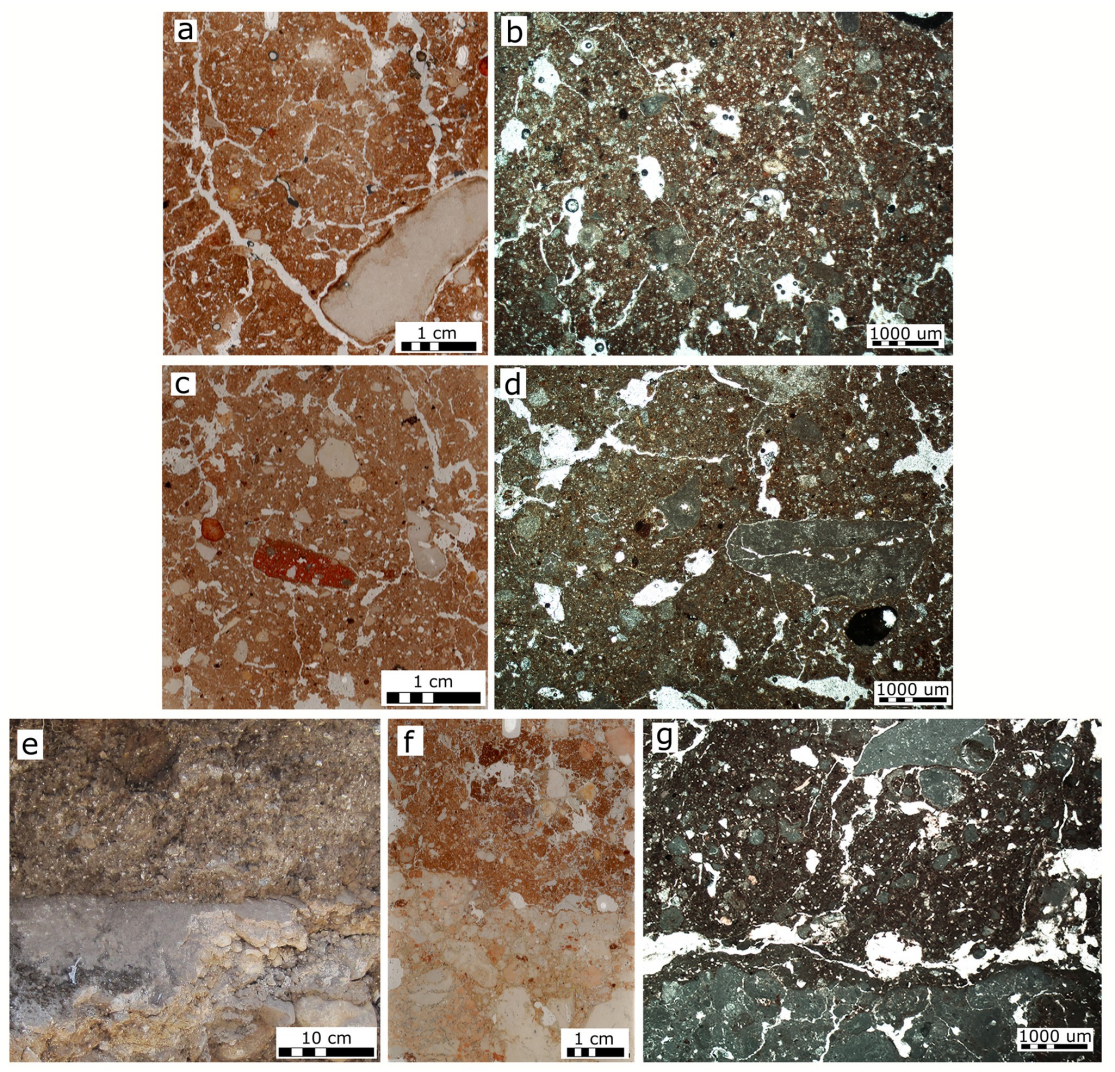
Results of micromorphology analysis. (a) Scan of thin section of a mudbrick from a Phase III wall (sample KAB-2450.5 in S2 Table). (b) Microphotograph of a Phase III mudbrick (sample KAB-2450.5 in S2 Table). (c) Scan of thin section of the fill deposit (5–11 cm) above the palaces’ Phase III floor (sample KAB-B2 in S2 Table). The red particles are pottery sherds. (d) Microphotograph showing the general appearance of the fill deposit shown in (c). (e) Field photo showing the Phase III plaster floor of Room 2553 and the fill deposit covering it. (f) Scan of thin section of the contact shown in (e) (sample KAB-2603.13 in S2 Table). (g) Microphotograph showing the contact between the floor and sediment lying directly on it shown in (f).

The mudbrick controls are composed of sub-rounded to rounded calcite nodules (200–500μm), chalk rock fragments (>500μm), and small amounts of mollusk shell and bone fragments incorporated within a brown groundmass composed of clay with quartz fine sand and silt. The clay component mainly possesses a speckled b-fabric, i.e., random specks of birefringence (an optical property of the material) in the clay component indicating origin from soil material. The related distribution between the coarse and fine fraction is single spaced porphyric (i.e., the coarse fraction is dispersed randomly and evenly within the fine fraction/groundmass). The overall microstructure is subangular blocky, exhibiting planar voids, vesicles, and channels (Fig 6a and 6b).

Phase III fill over the floors is comprised of the same main components as those identified in the mudbricks, as well as coarse pottery and bone fragments. A main difference in the fine fraction, in comparison to the mudbricks, is higher abundance of calcite, reflected by a mainly crystallitic b-fabric (i.e., birefringence originating from the presence of calcite rather than clay, indicating a mixture of calcitic components within the soil-derived mud brick material). The overall microstructure is subangular blocky. Voids include planes, vesicles, and channels (Fig 6c and 6d).

Specific attention was paid to the contact of the Phase III fill with the floor (Fig 6e and 6f). The floor is composed of pressed chalk clasts of variable sizes with no preferred orientation. The deposit just above the plaster floor is not laminated and does not include rolling pedofeatures or other features typical of water-lain deposits or mud slurry (hyperconcentrated) flows (Fig 6g). There is no compositional or microstructural difference between fill deposits lying directly on the floor (Fig 6g) or further above it (Fig 6d); both appear quite similar to the mudbrick and contain fragments from wall plaster (Fig 6b).

The mudbricks are composed of what appears to be local natural deposits, containing a negligible amount of anthropogenic debris, while the fill above the Phase III floor incorporates anthropogenic debris and a higher presence of calcite in both the groundmass and coarse fraction. The composition of the fill thus reflects a mixture of mudbrick material, calcitic (most probably wall) plaster, and anthropogenic debris from human activity during the palace’s last phase.

#### FTIR

Results of infrared analysis show that sediment samples from the Phase III fill deposits and from the mudbrick controls produced very similar spectra (S2 Fig and S1 Table), dominated by calcite and clay. Quartz, identified by the main absorbance band at 1080 cm^-1^ (which is at times obscured by the main absorbance band of clay) and a doublet at 778 and 798cm^-1^ [20], is present in low quantities. Carbonated hydroxyl apatite is present in minor amounts in several fill deposit samples, probably originating from bone fragments and/or decayed organic matter.

None of the sediment samples collected from the fill deposits above the palace’s Phase III storage room plaster floors show any indication of clay minerals being exposed to temperatures equal to or above 500°C, following [49] and [52]. Lack of evidence for high temperatures is also demonstrated through the ν_2_/ν_4_ ratios of calcite, showing that this mineral is geogenic (following [50]). Both calcite and clay results indicate that fire-induced destruction is highly unlikely.

## Discussion

A comprehensive examination of the Tel Kabri MB II palace was carried out in order to shed light on the reason(s) for its demise during what appears to be a very prosperous period in its history. These are discussed below, in light of additional factors that could have led to the abandonment of the site.

Pollen records from the southern Levant indicate a relatively wet period between 1750–1550 BCE [53, 54], correlated to a period of high lake levels in the Dead Sea [55]. This agrees with climatic conditions in central Europe during this time (e.g. [56]). Therefore, it seems that there was no extreme environmental crisis, or vast fluctuations in the climate during the MB II, at the time of abandonment.

Economic decline also seems not to have been a factor. On the contrary, a significant renovation program was implemented in the palace during its last constructional phase (site stratigraphic Phase III), just a short time before its end, showing that considerable means were still available at the time [32, 51].

Another indicator of wealth towards the palace’s end was uncovered in the large storage rooms, which contained pithoi that were filled with at least 4000 liters of wine when the palace was destroyed [33]. That quantity of wine would have had an estimated value of 625 silver shekels, a very high sum in a society where a worker’s salary was one shekel per month and a sheep cost 1.5 shekels [33].

Furthermore, the palace seems to have had several traits that strengthened its environmental resilience: zooarchaeological finds are consistent with a diverse and non-specializing animal economy using different ecological niches within the nearby territories without creating excessive environmental stress ([57] and references therein). The use of fuel for commodities consumed by the palace was also evaluated. A study of the plaster floors [58] concluded that most of their volume was prepared from pulverized chalk, while only a few localities included very thin (less than 1 cm) superimposed lime plaster surfaces. Similarly, the pithoi used in the wine storage rooms were shown to be fired at low temperatures not exceeding 600°C, in contrast to findings from other Bronze Age palatial workshops [59]. Taken together, both studies indicate that demands on local wood fuel supplies were not excessive, as pyrotechnology was conducted on a rather fuel-conservative scale. Kabri seems to have been maintaining a sustainable relationship with its environment.

Finally, it seems to us that the destruction of Kabri is unlikely to have been caused by violent human activity. There are no visible signs of conflagration, no weapons such as arrows like those uncovered in the destruction layer of Ugarit (ca. 1190 BCE–[60, 61]) that would indicate a battle, nor any unburied bodies related to combat. No hoards that would indicate preparation for a siege or an organized abandonment have been found, nor mass graves that indicate pandemic fatalities. Additionally, the MB II is a peaceful period in terms of Egyptian military activity. The demise of Kabri occurs during the Second Intermediate Period in Egypt and thus postdates the earlier Middle Kingdom incursions into Canaan, such as that of Khu-Sebek, which took place during the days of Senusret III in the 19th century BCE. At the same time, the abandonment of the site predates the renewals of Egyptian campaigns during the 18^th^ dynasty, beginning with Ahmose, ca. 1540–1530 BCE [62]. Furthermore, there seems to be an overall decrease in the level of intra- and inter-group violence in Canaanite society, which is reflected in a dramatic decrease in the number of warrior tombs as well as weapons in burials from MB I to MB II [63].

The results of the above-mentioned studies strongly suggest that scenarios of gradual climatic, environmental, or economic collapse, or a violent conquest, are unlikely causes for the demise of the Kabri palace. One of the main questions related to the type of destruction that befell the palace is whether floor deposits were buried quickly or gradually; the former would imply intentional collapse caused by humans or an earthquake, while the latter would be indicative of gradual disintegration following an abandonment of the palace.

Results of the micromorphological study show that the fill above the Phase III floor is a mixture of construction materials and anthropogenic debris with a massive chaotic structure. While evidence for bioturbation is present (e.g., channel voids), it is unlikely that it would have completely obliterated evidence for mud slurries, as these are found in other archaeological deposits from various periods across Israel despite bioturbation. Overall, absence of evidence for water-lain or mud slurry deposits indicates that construction materials did not accumulate slowly and sequentially, as would be expected following prolonged abandonment [64–66]. In addition, the excellent state of bone preservation (especially the amount of collagen preserved within the bones) also supports the scenario of a rapid collapse and burial [59].

In our opinion, the results of the microarchaeological study present compelling evidence that the collapse of the palace was not gradual. The chaotic arrangement of post Phase III floor deposits, together with lack of mud slurry deposits, implies a rapid collapse rather than the slow accumulation of degraded mudbrick material from standing walls or ceilings of an abandoned structure. This would also explain the jars in Room 2440 that were found lying on their side but which retained their shape without being crushed, which would happen if they were initially filled with sediment from the collapsing walls/ceiling and then fell over.

The rapid collapse and quick burial without conflagration, combined with the geological setting of Tel Kabri, raises the possibility that one or more earthquakes could have destroyed the walls and the roof of the palace without setting it on fire. Our reexamination of archaeological data presented above has revealed off-fault stratigraphic and structural elements, PEAEs [11], that point to damage resulting from at least one seismic event (i.e., an earthquake). These include broken and/or tilted and warped floors, wall plaster buildup, and fallen and broken objects that sometimes have similar spatial orientation. Perhaps the most telltale sign is the presence of the large trench that cuts across the palace. It is cut in turn by an unexcavated Iron Age pit (Fig 2) located west of the northern room of the Orthostat Building and east of the southeast corner of the Northern Storage Complex, which provides a terminus ante quem. Therefore, the trench cannot be a modern-day feature, as initially thought, but must instead be pre-Iron Age.

Since it was discovered during the 2019 excavations that the trench was filled with debris solely from the final phase of the palace (Phase III), we propose that it may represent a ground rupture resulting from earthquake activity. We note also that the trench runs parallel to a number of prominent, and otherwise unexplained, features in the palace. These include the folded floors of Room 2440 and associated displaced walls (e.g., Fig 4c and 4d) as well as the offsets and displacements recognized in the Orthostat Building (Room 2372, Fig 2). Moreover, both the trench and the features mentioned here all run parallel to the trace of the Kabri fault. Future excavations across the trench may add further data as to the nature and timing of this feature.

Having assessed the other possibilities as being substantially less likely, the mounting evidence as presented above leads us to suggest that at least one earthquake substantially damaged the site towards the end of the MB II, at around 1700 BCE. Data indicate that the damage to the palace, and the (rapid) collapse of at least parts of it, occurred prior to the subsequent abandonment of the site by the inhabitants. After several centuries of successful occupation and in a period that appears to have otherwise been quite prosperous, it must have been a very severe quake that not only damaged the palace, but also somehow destroyed the ability to actually continue living at the site. While Tel Kabri is not situated along or near the main plate boundary (the Dead Sea fault), it is nonetheless located along the trace of a potentially active fault (the Kabri fault) and in the vicinity of many others. Thus, it is certainly possible that an earthquake could have destabilized an already susceptible area with very high groundwater (as evident by the four springs in the area) and anomalously high potential ground motion amplification, such as at Tel Kabri.

The question then arises as to what could have caused the inhabitants’ decision not to continue to occupy the site? An earthquake could have interrupted the flow of water from the local springs, similar to what has been suggested for the site of Pella, Jordan [67], coincidentally (or not) around the same time. If so, then the abandonment of the site by the inhabitants of Kabri may have been the result of the combined effect of damage to buildings and to their water supply. While the former could be repaired, the latter could be terminal.

Even if the water supply were not affected, such physical damage to buildings and structures from an earthquake could also have caused further harm to subsistence, essential infrastructures, and social cohesion. This, in turn, could have led to a loss of legitimacy of governmental institutions in the eyes of the people (e.g. [68]). Compounding of earthquakes with other natural and anthropogenic calamities was familiar to the people of the ancient Near East [69]. The effect is best described in the cuneiform text SMN 3180 from Nuzi (14th century BCE), containing a list of omens describing the calamities that will occur if an earthquake hits during different months of the year. Some omens predict famine, pestilence, and destruction of settlements and land, while other calamities are connected with collapse of social order, e.g., revolt against the king, invasion, the loss of power of the ruler, or loss of the palace. For example, in the omen for the month of Tebet, it is written: "If the earth quakes in Tebet, the palace and the land will go to ruin” [70, 71]. The connection to loss of social cohesion can also be seen in the first millennium BCE Akkadian commentaries, predicting a rebellion as a result of an earthquake [72]. Thought speculative, it is not unconceivable that the physical damage apparently caused by the earthquake at Kabri, particularly in the form of the very visible trench in the earth that now crossed the palace from side to side, may have been regarded as a sign of divine displeasure with the rulership and populace, also deterring them from rebuilding.

In sum, the results of micro-geoarchaeological indicators used in this study point to a society that was aware of its surroundings and operated in a sustainable way with respect to the environment, as evident by the economic use of fuel expressed by firing of pottery at relatively low temperatures [59] and the small volumes of pyrogenic lime plaster [58]. This is supported by studies that showed that the population at the time focused on low volume, high value local produce, such as spiced wine and low intensity livestock production typical of mixed agriculture [33, 57]. In addition, micro-geoarchaeological indicators presented in the current study show that the palace was probably not destroyed by fire, nor was it left to slow, natural decay. Collapse of mudbrick walls along with wall and roof plaster was quick.

### Conclusions

The above sections presented both micro-geoarchaeological data and macro field observations to assess the possible reasons for damage to the MB II palace at Tel Kabri ca. 3700 years ago and the subsequent abandonment of the site for centuries.

The integrated approach used in this study allowed us to take into account macroscopic indicators for earthquake damage and collapse in architectural features and artifacts (PEAEs) as well as micro-geoarchaeological indicators for the rapid mode of deposit accumulation without evidence for fire. Furthermore, by examining previous studies, we argued that other possible scenarios for the demise of MB II Kabri, such as economic collapse, are far less likely than a hypothesis that the site was severely damaged by an earthquake, which led to its abandonment soon thereafter. In closing, we would suggest that the integrated multi-scalar methodological approach presented here can be applied at other archaeological sites, where it can serve to test and/or strengthen cases of possible earthquake damage and destruction.

## Supporting information

S1 Fig Field photographs demonstrating field sampling of bulk sediments. (a) Wall W2450 and its surrounding. The blue tags indicate bulk sample positions. a: mud bricks in the wall; b: white plaster lining the wall; c: fill deposits next to the wall associated with several pithoi on the floor next to the wall. Note the white-speckled nature of the fill deposits, due to abundant plaster fragments. (b) Field photograph exemplifying sampling of block samples in Room 2520. a: Phase III floor; b: ceramic walls of a storage jar lying on the floor and sectioned in situ; c: fill deposit.
(TIF)

S2 Fig Infrared spectra of (a) representative sediment sample of the Phase III fill deposits, dominated by calcite (blue) and clay (red), with minor absorptions of quartz (green), and (b), representative mud brick sample from Wall W2450 showing higher amount of clay relative to calcite in comparison to the fill deposits; the reason is presence of wall/ceiling plaster fragments in the former. The position of the main clay absorbance band at 1033 cm-1 in both samples, associated with presence of absorbance bands at 532, 912, 3621 and 3696 cm-1, indicates the clay did not experience heat above 500°C.
(TIF)

S1 Table List and field description of bulk sediment samples collected across the Phase III Southern Storage Complex in Area D-West at Tel Kabri. Results of infrared (FTIR) analysis per sample are presented as well (n.a. not analyzed). Calcite and clay spectra do not indicate exposure to heat above 500°C. > indicates higher amounts of a mineral relative to the other. Trace amounts of minerals are indicated in parentheses. CHAP: carbonated hydroxyl apatite (from bones and/or organic matter).
(DOCX)

S2 Table List and field description of block sediment samples collected across the Phase III Southern Storage Complex in Area D-West at Tel Kabri. (DOCX)
