# Supplementary figures and images for: Earthquake damage as a catalyst to abandonment of a Middle Bronze Age settlement: Tel Kabri, Israel
Source: PLoS One. 2020 Sep 11;15(9):e0239079. doi: 10.1371/journal.pone.0239079 (PMC7485796; doi:10.1371/journal.pone.0239079)

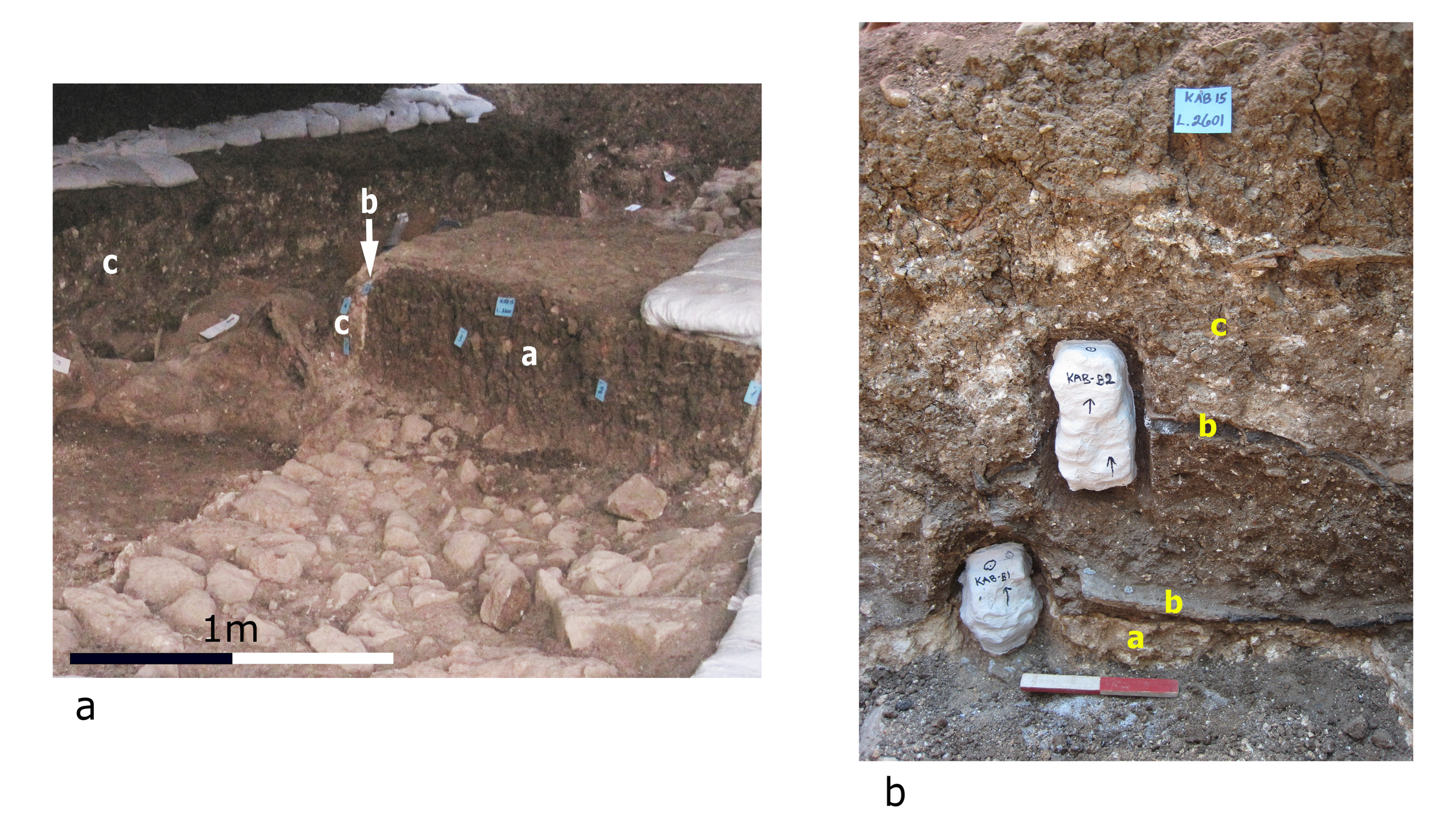

Supplement: S1 Fig — (a) Wall W2450 and its surrounding. The blue tags indicate bulk sample positions. a: mud bricks in the wall; b: white plaster lining the wall; c: fill deposits next to the wall associated with several pithoi on the floor next to the wall. Note the white-speckled nature of the fill deposits, due to abundant plaster fragments. (b) Field photograph exemplifying sampling of block samples in Room 2520. a: Phase III floor; b: ceramic walls of a storage jar lying on the floor and sectioned in situ; c: fill deposit. (TIF) [file pone.0239079.s001.tif]

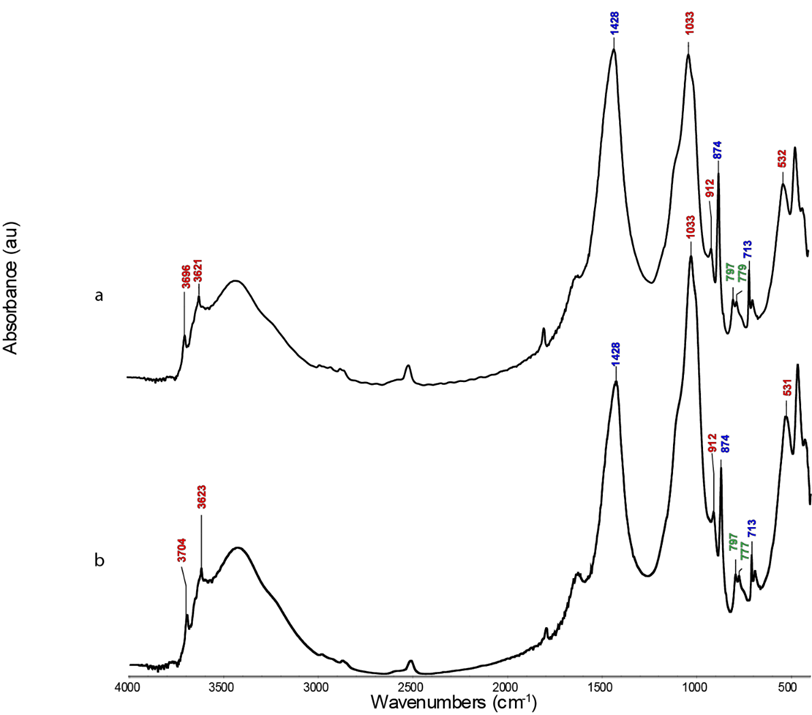

Supplement: S2 Fig — The position of the main clay absorbance band at 1033 cm-1 in both samples, associated with presence of absorbance bands at 532, 912, 3621 and 3696 cm-1, indicates the clay did not experience heat above 500°C. (TIF) [file pone.0239079.s002.tif]
