# Supplementary material for: Earthquake damage as a catalyst to abandonment of a Middle Bronze Age settlement: Tel Kabri, Israel
Source: PLoS One. 2020 Sep 11;15(9):e0239079. doi: 10.1371/journal.pone.0239079 (PMC7485796; doi:10.1371/journal.pone.0239079)
Supplement: S1 Table — Results of infrared (FTIR) analysis per sample are presented as well (n.a. not analyzed). Calcite and clay spectra do not indicate exposure to heat above 500°C. > indicates higher amounts of a mineral relative to the other. Trace amounts of minerals are indicated in parentheses. CHAP: carbonated hydroxyl apatite (from bones and/or organic matter). (DOCX) [file pone.0239079.s003.docx]

| **Sample number** |  | **Field description** | **FTIR results** |
| --- | --- | --- | --- |
| **Controls** |  |  |  |
| KAB15.L2600-2 |  | Mud brick (locus 2491) | Clay > calcite |
| KAB15.L2600-3 |  | Mud brick (locus 2491) | Clay > calcite |
| KAB16.L2450-1 |  | Mud brick control sample from the eastern face of wall 2450 | Clay > calcite, (quartz) |
| KAB16.L2450-2 |  | Mud brick control sample from the southern face of wall 2450 | Clay > calcite, (quartz) |
| **Phase III fill above floors** |  |  |  |
| KAB15.L2600-5 |  | Orange mud brick (?) | Calcite > clay |
| KAB15.L2600-6 |  | Brown-grey "fill" | Calcite > clay |
| KAB15.L2601-1 |  | Bright white fragment | Calcite |
| KAB15.L2601-2 |  | Dull white fragment | Calcite |
| KAB15.L2601-3 |  | Topmost part of plaster floor | Calcite |
| KAB15.L2601-4 |  | Bright white within collapse | n.a. |
| KAB15.L2601-5 |  | Bright white within collapse | Calcite |
| KAB15.L2601-6 |  | Bright white within the jar | n.a. |
| KAB15.L2601-7 |  | Dark brown sed. below jar | Clay > calcite |
| KAB15.L2601-8 |  | Brown sediment within jar | Calcite = clay |
| KAB15.L2601-9 |  | Brown-grey sed. above jar | Calcite > clay |
| KAB15.L2601-10 |  | Reddish-brown agricultural soil | Calcite > clay |
| KAB15.L2601-11 |  | Yellowish-grey material in collapse | n.a. |
| KAB16.L2602-1 |  | Light brown sediment- general sample of archaeological deposit | Calcite > clay, (quartz, CHAP) |
| KAB16.L2602-2 |  | Reddish brown sediment- general sample of possible mud brick collapse | Calcite > clay, (quartz) |
| KAB16.L2602-3 |  | Dark brown sediment- general top-soil sample | Clay > calcite, (quartz, CHAP) |
| KAB16.L2602-4 |  | Brownish gray sediment above floor 2477 | Calcite > clay, (quartz, CHAP) |
| KAB16.L2602-5 |  | Grayish sediment from within a pot in the section | Calcite > clay, (quartz, CHAP) |
| KAB16.L2602-6 |  | Grayish sediment above the pot | Calcite > clay, (quartz, CHAP) |
| KAB16.L2602-8 |  | Reddish patch maybe mud brick | Calcite = clay, (quartz, CHAP) |
| KAB16.L2602-10 |  | Reddish sediment maybe mud bricks | Calcite > clay, (quartz, CHAP) |
| KAB16.L2602-12 |  | Very dark thin layer in top part of the section | Clay > calcite, (quartz, CHAP) |
| KAB16.L2603-1 |  | Light brown sediment directly above floor 2553 | Calcite > clay, (quartz, CHAP) |
| KAB16.L2603-2 |  | Light brown sediment above pottery with small white stones | Clay > calcite, (quartz) |
| KAB16.L2603-3 |  | Reddish brown sediment maybe mud brick | Calcite = clay, (quartz, CHAP) |
| **Plaster** |  |  |  |
| KAB15.L2600-1 |  | Wall plaster | Calcite |
| KAB15.L2600-4 |  | Wall plaster | Calcite |
| KAB15.L2600-7 |  | Floor plaster in room 2520 | Calcite |
| KAB15.L2600-8 |  | Floor plaster in room 2520 | Calcite |
