# Supplementary material for: Earthquake damage as a catalyst to abandonment of a Middle Bronze Age settlement: Tel Kabri, Israel
Source: PLoS One. 2020 Sep 11;15(9):e0239079. doi: 10.1371/journal.pone.0239079 (PMC7485796; doi:10.1371/journal.pone.0239079)
Supplement: S2 Table — (DOCX) [file pone.0239079.s004.docx]

| **Sample number** | **Field description** |
| --- | --- |
| **Controls** |  |
| KAB-2450.5 | Sample of mudbrick wall 2450 |
| KAB-2450.5a | Second sample of mudbrick wall 2450 |
| KAB-2450.6 | Third sample of mudbrick wall 2450 |
| **Phase III fill above floors** |  |
| KAB-B1 | Floor 2520+sediment above floor, 10cm north of jar |
| KAB-B2 | Fill within jar 2526.1+in situ body sherd of jar+collapse 2505 above jar |
| KAB-2603.13 | Floor 2553+sediment above floor. |
